# The Impact of the 2013 Eastern China Smog on Outpatient Visits for Coronary Heart Disease in Shanghai, China

**DOI:** 10.3390/ijerph13070627

**Published:** 2016-06-23

**Authors:** Fang Huang, Renjie Chen, Yuetian Shen, Haidong Kan, Xingya Kuang

**Affiliations:** 1 Department of Occupational Medicine, Yangpu Hospital, Tongji University School of Medicine, Shanghai 200090, China; huangfang19861986@163.com (F.H.); 13641813262@163.com (Y.S.); 2 Department of Occupational Medicine, Yangpu District Central Hospital, Shanghai 200090, China; 3 Institute of Occupational and Environmental Medicine, Medical School, Tongji University School of Medicine, Shanghai 200090, China; 4 School of Public Health, Key Lab of Public Health Safety of the Ministry of Education, Fudan University, Shanghai 200032, China; crj_1986@163.com (R.C.); haidongkan@gmail.com (H.K.)

**Keywords:** smog, air pollution, coronary heart disease, outpatient visits, time-series

## Abstract

There have been relatively few opportunities to examine the cardiovascular effects of an extreme air pollution event in China. We aimed to examine the impact of the 2013 Eastern China Smog occurring from 2 to 9 December 2013, on outpatient visits for coronary heart diseases (CHD) in a typical hospital in Shanghai, China. We used the over-dispersed, generalized additive model to estimate the relative risk (RR) of the 2013 Eastern China Smog on the outpatient visits by comparing the smog period (2–9 December 2013; 8 days) to the non-smog period (1 November–1 December 2013, and 10 December–28 February 2014; 112 days). This model also controlled for time trends, days of the week, holidays, and meteorological factors. A stratification analysis was performed to estimate sex- and age-specific RRs. The daily average PM_2.5_ (fine particulate matter with an aerodynamic diameter less than 2.5 μm) concentrations during the smog period were 212 μg/m^3^, which were three times higher than during the non-smog period (76 μg/m^3^). The smog in Eastern China in 2013 was significantly associated with an increased risk of outpatient visits for CHD. For example, the RR was 1.18 (95% CI: 1.04, 1.32) on lag 0 day. There were similar effects on males and females. Our analyses provided preliminary evidence that smog constituted a significant risk factor of CHD in China.

## 1. Introduction

Ambient air pollution is the largest single environmental risk factor that caused more than 3 million premature deaths in 2013 worldwide [1], particularly in developing countries with rapid industrialization and urbanization, such as China. The unprecedented economic growth of China in the last three decades has resulted in serious air pollution problems on both local and regional scales. Megacities in China, such as Beijing and Shanghai, have suffered from frequent smog events, with the daily concentrations of fine particulate matter far beyond the Chinese air pollution standard of 75 μg/m^3^ [2,3]. For example, in December 2013, a severe air pollution event with low visibility, long duration, and extensive geographic coverage occurred in eastern China with the PM_2.5_ reaching unprecedented high levels, which was named as the “2013 Eastern China Smog” thereafter.

Dozens of epidemiological studies have demonstrated that day-to-day variations in ambient air pollution are positively associated with mortality and hospitalizations for cardiopulmonary diseases [4,5,6,7]. However, few studies have evaluated the health impacts of a smog period, in which the exposure–response relationship may be different from that on general days. Understanding the excessive health risks of a smog period is crucial to developing public health policies.

As the largest city in China, Shanghai is experiencing poor air quality compared with other mega cities in North America and Western Europe. Therefore, the objective of this study was to evaluate the impacts of the 2013 Eastern China Smog occurring from 2 to 9 December 2013, on outpatient visits for CHD in a large hospital in this city. CHD was selected because of its high sensitivity to air pollution reported in previous studies [8,9,10].

## 2. Materials and Methods

### 2.1. Data Collection

To allow for a comparison between smog days and non-smog days, we collected the daily numbers of outpatient visits for CHD during the period between 1 November 2013 and 28 February 2014, from Yangpu Hospital, Tongji University. This is a district-level hospital located in a central urban district (Yangpu) of Shanghai. Unlike the top hospitals in Shanghai that serve a large proportion of nonlocal patients, Yangpu Hospital mainly serves permanent residents in this district, which may be representative to some extent of the Shanghai population. In recent years, this hospital had a yearly total visit of approximately 1.5 million patients in the outpatient and emergency departments. We derived data on CHD events from the outpatient department. The diagnosis of CHD was completed by physicians based on the patients’ symptoms, inquiries, complaints, and findings on medical inspections. Then, the physicians submitted standard electronic documents through the internal computer network, which included basic information (e.g., name, sex, address, and telephone number), as well as the primary diagnosis and treatment. We then aggregated the daily number of visits of the patients whose primary diagnosis was CHD from the outpatient department. We excluded the patients whose permanent addresses were out of this district. The Institutional Review Board at the School of Public Health, Fudan University, approved the study protocol (No. 2012-03-0324) with a waiver of informed consent. Data were analyzed at aggregate level, and no participants were contacted.

Daily (24-h) average concentrations of air pollutants, including PM_2.5_, sulfur dioxide (SO_2_), nitrogen dioxide (NO_2_), carbon monoxide (CO), and ozone (O_3_), were collected at a state-controlled monitoring station in the same district as the hospital, approximately 1 km away from the hospital. These pollutants were measured based on the methods of tapered element oscillating microbalance, ultraviolet fluorescence, chemiluminescence, light absorbance, and ultraviolet fluorescence, respectively. For the calculation of 24-h averages, at least 75% of the hourly concentrations had to be available in a single day. The location of this monitor was mandated not to be in the direct vicinity of traffic, industrial pollution, or other local pollution sources and not to be influenced by buildings or large housing emitters, such as coal-, waste-, or oil-burning boilers; furnaces; and incinerators. Therefore, the air pollutant measurements represent the levels of exposure to urban background air pollution in the general population of this district.

To allow for the adjustment of weather conditions, we collected daily ambient temperatures and relative humidity from the Shanghai Meteorological Bureau.

### 2.2. Statistical Analysis

A time-series approach is often used to analyze the association between air pollution, extreme climate exposure and the aggregated counts of a health outcome [10,11,12]. To be specific, we used a generalized additive model (GAM) with a quasi-Poisson link to quantify the impacts of this smog event on the outpatient visits for CHD [5,8,9]. A smog period was introduced as a dummy independent variable with 1 denoting the smog day and 0 denoting the non-smog day. The risk was estimated by comparing the smog period (2–9 December 2013; 8 days) to the non-smog period (1 November–1 December 2013, and 10 December–28 February 2014; 112 days). The three periods were in the same season to reduce confounding from unmeasured time-varying factors. We also controlled for several covariates: (1) a calendar day term with 3 degrees of freedom (*df*) in the natural splines; (2) days of the week; (3) a dummy variable of holidays; and (4) 3 *df* in the natural splines of daily mean temperatures and relative humidity.

To analyze the potential lag pattern in the effects of the smog period, we fit the above basic model with single lags of 0 to 7 days. We further performed a stratification analysis by age (grouped by 5–64 years old and 65 years old or above) and sex to explore their possible modifications in the association between a smog event and CHD.

All of the statistical tests were two-sided, and the values of *p* < 0.05 were considered statistically significant. The statistical analyses were conducted by R 3.3.0 using the mixed GAM computation vehicle (MGCV) package (R Foundation for Statistical Computing, Vienna, Austria).

## 3. Results

Table 1 shows the descriptive statistics of the outpatient visits for CHD, air pollutants, and weather conditions from 1 November 2013 to 28 February 2014. On average, there were 141 outpatient visits for CHD per day during the entire study period (169 visits in the smog period, and 139 visits in the non-smog period). The daily average concentrations of PM_2.5_, SO_2_, NO_2_, CO, and O_3_ were 85 μg/m^3^, 33 μg/m^3^, 56 μg/m^3^, 0.71 mg/m^3^, and 45 μg/m^3^, respectively. The weather was cold and dry during the study period. The daily 24-h average PM_2.5_ concentrations during the smog period were 212 μg/m^3^, three times higher than during the non-smog period (76 μg/m^3^). The daily 24-h average concentrations of SO_2_, NO_2_, and CO were also higher during the smog period than the non-smog period (48 μg/m^3^ vs. 32 μg/m^3^, 102 μg/m^3^ vs. 54 μg/m^3^, and 1.15 μg/m^3^ vs. 0.68 μg/m^3^, respectively). However, the daily 8-h average concentrations of O_3_ were lower during the smog period than the non-smog period (34 μg/m^3^ vs. 46 μg/m^3^, respectively). A spearman correlation analysis showed that PM_2.5_, SO_2_, NO_2_, and CO were strongly correlated with each other (r = 0.70 − 0.88), but they were negatively related with O_3_. These air pollutants were weakly or negatively related with the weather conditions (Table 2).

Table 3 summarizes the RRs for CHD outpatient visits by comparing the smog period to the non-smog period. The RRs were statistically significant at lags 0 to 3 days. For example, the smog was significantly associated with the 17% (95% CI: 4%, 28%), 13% (95% CI: 0%, 25%), 13% (95% CI: 0%, 25%), and 16% (95% CI: 4%, 27%) increases in the outpatient visits for CHD on lags of 0, 1, 2, and 3 days, respectively. Thereafter, the RRs turned out to be statistically insignificant, but they were still positive around 1. According to the results of the stratification analysis, the risk estimates were similar among males and females and were somewhat higher among people younger than 65 years.

## 4. Discussion

This time-series study demonstrated a significant increased risk of CHD associated with the 2013 Eastern China Smog in Shanghai, China. Our findings may have important implications for environmental policies in China, and for the Chinese government to take the necessary steps to protect public health in general and sensitive populations in particular.

In this study, the 2013 Eastern China Smog was associated with a 17% (95% CI: 4%, 28%) increase in outpatient visits for CHD on the concurrent day in Shanghai. Our results were generally consistent with previous epidemiological studies regarding haze pollution. For example, Zhang et al. showed that the daily incidence of acute cardiovascular diseases was higher on the haze days than on the non-haze days, and there was a positive relationship between cases of acute cardiovascular diseases and the number of haze days [13]. The authors further estimated an 11% (95% CI: 4%, 19%) increase in daily hospital admissions for cardiovascular diseases during a haze episode in Guangzhou, China [14].

In the present analysis, we did not observe a “harvesting effect” because there was no significant decline below 1 for the RRs in extended lags, suggesting that the smog may immediately lead to CHD events. The significant association between the smog and CHD outpatient visits was supported by previous epidemiological studies and mechanistic studies. A number of epidemiological studies have shown the association between the short-term exposure to PM_2.5_ and the increased mortality or incidence of CHD, myocardial infarction, heart failure and cardiac arrest [8,15,16,17]. Several biological pathways have been suggested to be responsible for the association between smog and CHD, including exaggerated inflammatory response, increased blood coagulability, altered cardiac autonomic nervous system, elevated blood pressure, and arrhythmia [4,18,19].

Few studies have evaluated the modification by sex and age groups in the associations between smog and health outcomes. We found similar effects of the smog period on both males and females. The existing evidence about between-sex differences is far from consistent [1,20,21,22]. It is unclear whether the observed differences are attributable largely to sex-linked biology, work-related exposure differences, or socially derived roles and activities [23]. We found a somewhat higher effect of the smog event on CHD among people younger than 65 years than those people older than 65 years. Similarly, Metzger et al. reported no evidence for an increased risk of cardiovascular diseases hospital admissions associated with PM_2.5_ among older adults compared with younger adults [10]. However, these results were contrary to most previous studies on PM_2.5_ and health outcomes [21,22]. This discrepancy between our results and most of the previous findings may be due to the imbalance of a number of confounding factors between the two groups, such as a history of CHD, co morbidities, and personal behaviors (e.g., dietary patterns, physical activities, tobacco smoking, and alcohol use). Another important explanation may be that young people have more outdoor exposure than older people, as the elders may be advised to stay indoors when a warning is released.

Our analyses had some limitations. First, as in most of the previous time-series studies, exposure measurement error and diagnosis bias were inevitable in this analysis. Further, this error might be more important due to the potential self-protection from air pollution, such as staying indoors and wearing masks. However, this was not a significant problem because we only used fixed-site measurements to define the smog period and non-smog period. Second, limited by the ecological design and the complex atmospheric conditions during the smog period, this study could not identify the causal driver of the increased CHD events in this period. Third, although the data from Yangpu Hospital may be representative to some extent of the Shanghai population, the extrapolation of our results should be interpreted with caution because no other hospitals were evaluated.

## 5. Conclusions

This time-series analysis provided preliminary evidence that the 2013 Eastern China Smog constituted a significant risk factor of CHD in Shanghai, China. Our results support that continuous and persistent efforts should be taken to improve ambient air quality in this country.

## Figures and Tables

**Table 1:** Descriptive statistics of outpatient visits for coronary heart diseases (CHD), air pollutants, and weather conditions from 1 November 2013 to 28 February 2014 (120 days in total), in this study.

Variables	Mean ± SD	Min	P25	Median	P75	Max
Outpatients for CHD	141.1 ± 77.2	11.0	62.5	159.0	199.5	272.0
Air pollutants (μg/m^3^)						
PM_2.5_	85.1 ± 65.7	7.0	41.5	65.0	111.5	461.0
SO_2_	32.6 ± 20.5	9.0	17.0	25.0	46.0	93.0
NO_2_	56.2 ± 25.9	13.0	38.5	50.0	72.0	128.0
CO (mg/m^3^)	0.7 ± 0.3	0.3	0.5	0.6	0.9	1.8
O_3_	45.1 ± 19.6	13.1	28.7	41.2	61.5	103.1
Weather conditions						
Temperature (°C)	8.6 ± 4.9	0.0	4.4	7.8	11.8	22.8
Humidity (%)	63.7 ± 13.8	29.0	55.8	63.5	73.0	92.0

**Table 2:** Spearman correlation coefficients between daily air pollutant concentrations and weather conditions.

Variables	SO_2_	NO_2_	CO	O_3_	Temperature	Humidity
PM_2.5_	0.78	0.80	0.88	−0.42	0.20	−0.21
SO_2_		0.83	0.70	−0.66	−0.06	−0.62
NO_2_			0.78	−0.54	0.21	−0.37
CO				−0.57	−0.10	0.03
O_3_					0.47	0.31
Temperature						0.24

**Table 3:** Relative risks (RRs) of outpatient visits due to CHD in smog period from 2 to 9 December 2013.

Lags	All		Male		Female		5–64 ys		≥65 ys	
	RR	95% CI	RR	95% CI	RR	95% CI	RR	95% CI	RR	95% CI
0 d	1.180	1.04, 1.32	1.174	1.01, 1.34 *	1.190	1.04, 1.33 *	1.230	1.06, 1.40 *	1.160	1.00, 1.31 *
1 d	1.140	1.00, 1.28 *	1.144	0.98, 1.30	1.130	0.99, 1.28	1.190	1.03, 1.35 *	1.110	0.96, 1.26
2 d	1.140	1.00, 1.28 *	1.160	1.00, 1.32 *	1.130	0.98, 1.27	1.200	1.03, 1.36^*^	1.120	0.97, 1.27
3 d	1.170	1.04, 1.31 *	1.160	1.01, 1.32 *	1.180	1.05, 1.32^*^	1.190	1.03, 1.35 *	1.170	1.02, 1.31 *
4 d	1.105	0.97, 1.24	1.095	0.93, 1.26	1.114	0.97, 1.26	1.072	0.90, 1.25	1.121	0.98, 1.27
5 d	1.063	0.92, 1.21	1.054	0.89, 1.22	1.071	0.92, 1.22	1.055	0.88, 1.23	1.067	0.92, 1.21
6 d	1.042	0.90, 1.19	1.044	0.88, 1.21	1.042	0.89, 1.19	1.024	0.84, 1.21	1.051	0.90, 1.20
7 d	1.019	0.88, 1.16	1.014	0.85, 1.17	1.024	0.88, 1.17	1.019	0.85, 1.19	1.020	0.87, 1.17

* *p* < 0.05.
